# Worth a local treatment? – Analysis of modern radiotherapy concepts for oligometastatic prostate cancer

**DOI:** 10.1186/s13014-018-1118-7

**Published:** 2018-09-21

**Authors:** M. Oertel, S. Scobioala, K. Kroeger, A. Baehr, L. Stegger, U. Haverkamp, M. Schäfers, H.-T. Eich

**Affiliations:** 1 Department of Radiation Oncology, Albert-Schweitzer Campus 1 A1, 48149 Muenster, Germany; 2 Department of Nuclear Medicine, Albert-Schweitzer Campus 1 A1, 48149 Muenster, Germany

**Keywords:** Prostate cancer, Lymph-node recurrence, IMRT, Salvage therapy

## Abstract

**Background:**

Prostate cancer (PCA) is the most-prevalent non-skin cancer in men worldwide. Nevertheless, the treatment of oligometastatic, especially lymph-node (ln) recurrent, PCA remains elusive. The aim of our study was to provide insights in radiotherapy (RT)-treatment of recurrent PCA exhibiting ln- or osseous (oss)-oligometastases.

**Methods:**

Between April 2012 and April 2017, 27 oligometastatic PCA patients (19 ln and 8 single oss) were treated with RT at our institution.

**Results:**

The metastasis-free survival (MFS) was 24.8 m (22.0–36.0 m) and 25.4 m (23.9–28.1 m) for the ln- and oss-subgroup resulting in 1-year MFS of 75.4 and 100% and 2-year MFS of 58.7 and 83.3% for ln- and oss-metastatic patients, respectively. Of notice, none of the recurrences for ln-patients was in the RT-field, constituting a local control of 100%.

Within the ln-group, pre-RT median-PSA was 2.6 ng/ml, median post-RT PSA was 0.3 ng/ml, which was significant (*p* = 0.003). Median biochemical-free survival (bfS) was 12.2 m. PCA that was initially confined to the prostate had a better bfS (*p* < 0.001) and MFS (*p* = 0.013). The oss-group had a median PSA of 4.9 ng/ml pre-treatment which dropped to a median value of 0.14 ng/ml (*p* = 0.004).

Toxicities were moderate, with only 1 case of III° toxicity. There were no deaths in the ln-group, thus overall survial was 100% here.

**Conclusion:**

Our study points out the feasibility of RT as a treatment option in recurrent PCA and demonstrates an excellent local control with a low-toxicity profile.

## Background

Prostate cancer (PCA) is the most prevalent non-skin cancer worldwide in men with over 63,000 newly diagnosed men each year in Germany and around 13,000 dying from it annually [1, 2]. It is biologically heterogenous with aggressive subtypes being contrasted by slow growing carcinoma with a long latency to diagnosis [3]. Histological carcinomas are found in autopsy-series from the 4th decade of life, whereas the diagnosis is at 71 of age (median) [2, 4]. In vivo diagnosis is achieved via digital-rectal exam (DRE), measurement of the prostate-specific antigen (PSA), transrectal ultrasound and systematic biopsy [5, 6] although even a combined approach with PSA-value and DRE may fail in detecting the disease [7]. After primary therapy (radical prostatectomy, radiotherapy (RT) as teletherapy or brachytherapy) PSA is recommended as a follow-up parameter [5, 6].

In case of recurrent PCA, functional imaging like PET-CT, has shown its value in identification or exclusion of localized tumor manifestations and may give guidance for directing RT and surgical therapy [5, 6, 8–14]. Also MRI may offer the possibility of precise and accurate cancer detection and target volume delineation in the PSA-relapse or metastatic situation [15].

National and international guidelines struggle to standardize treatment strategies in case of lymph-node (ln) metastasis after primary therapy [5, 6] focusing on systemic androgen-deprivation-therapy (ADT), although this treatment is known to be associated with considerable side effects such as diabetes, cardiovascular morbidity, decrease in bone density with danger of fracture, sexual dysfunctions and onset of depression [16, 17].

Anyhow, RT has shown promising results and may efficiently prolong the time till the initiation of ADT [8–11, 13, 18–25]. These studies included only limited patient numbers and were partly conducted retrospectively, calling for further investigation. In a more broad definition, these patients reveal an “oligometastatic state”, a term coined by Hellmann and Weichselbaum defining a disease state in which a low number of metastases with limited malignant potential exist, putatively without microscopic ubiquitous dissemination, forming an intermediate state between localized and generalized form of disease [26]. This concept implies a limitation of metastatic spread to one or a only some organs due to an impaired/undeveloped ability for further progression and highlights a continuum of biologic behavior in the development of cancer [26]. In the setting of oligometastases, local therapy (RT, operation) may be of use [23, 24, 27].

In the present study, we have analyzed the use of RT as an individual treatment approach for men with ln recurrent PCA. As a control group, we established a subcollective of single osseous metastasis (oss), thus also being oligometastatic. We demonstrate use and feasibility of image-guided RT with modern RT techniques and could present an effective treatment with high local control and tolerable toxicity.

## Methods

### Patients

Between April 2012 and April 2017, 19 patients with ln recurrent PCA were treated with RT at our institution. Median age at initial diagnosis was 60.6 years (y) (48.1–79.1 y) and 66.5 y (57.7–78.3 y) for ln- and oss-group, respectively. Further tumor characteristics are provided in Table 1. Primary therapy consisted of radical prostatectomy followed by post-operative or salvage RT in all but 1 case.

**Table 1:** Initial characteristics of the study patients. A: ln-metastatic patients; B: osseous metastases

A)			B)		
Age at inital diagnosis	median	60.6	Age at inital diagnosis	median	66.5
	maximum	79.1		maximum	78.3
	minimum	48.1		minimum	57.7
T	T2b	1	T	T2c	2
	T2c	5		T3a	1
	T3a	9		T3b	5
	T3b	4	N	N0	5
N	N0	16		N1	2
	N1	3		missing	1
M	M0	13	M	M0	4
	MX	1		missing	4
	missing	5	Gleason	7	3
Gleason	6	1		8	2
	7	10		9	3
	9	6	R-status	R0	2
	10	1		R1	3
	missing	1		missing	3
R-Status	R0	11			
	R1	6			
	missing	2			

At a median time of 5.4 y (0.5–15.6 y) or 6.7 y (2.2–16.6 y) after primary therapy, at a median age of 67.9 y (49.9–83.3 y) or 74.4 y (64.8–82 y) for ln- or oss-metastatic patients respectively, metastatic spread was diagnosed. The ln recurrence occurred in regionary (8), distant (5) or both ln-stations (6) (Table 2). Regionary lymph nodes were defined as pelvic ln below lumbal vertebra 5, marking the field border of a potential whole pelvic RT. We further simplified categories to distinguish between patients with regionary and distant (also involving combined occurrence of regionary ln) ln-regions. In the majority of patients 1–2 ln were involved. In the case of ≥3 metastases, a localized distribution was essential to define a RT volume, a disseminated metastatic situation being an exclusion criterion of our study. Diagnosis was achieved with functional imaging in 17/19 patients (17 PET-CT (2 C^11^-Choline; 15 Ga^68^-prostate-specific membrane antigen (PSMA)) and morphological via CT/MRI in 2 cases.

**Table 2:** Characteristic of diagnosis for the ln-recurrences (A) and single-osseous (B) metastases

A)	
Age at ln diagnosis	
median	67.9
maximum	49.9
minimum	83.3
region	
regionary	8
distant	5
regionary and distant	6
number of ln	
1	8
2	2
≥3	9
imaging	
PET-CT (Ga^68^-PSMA)	15
PET-CT (C^11^-Cholin)	2
CT/MRT	2
B)	
Age at metastases diagnosis	
median	74.4
maximum	82.0
minimum	64.8
imaging	
PET-CT (Ga^68^-PSMA)	4
PET-CT (C^11^-Cholin)	1
SPECT (Tc^99m^)	1
CT/MRT	1
CT	1

All patients were reviewed interdisciplinary and were counseled about RT as an individual treatment approach in their oncological situation. Informed consent was given.

Twenty patients received an ADT during treatment (13 in the ln-group, 7 in the oss-group).

### Radiotherapy

Overall, 37 RT-series were conducted in the 29 ln-group and 8 in the oss-subgroup patients with the anatomic regions shown in Table 1. Concentrating on the ln-group, a salvage RT of the prostate bed was part of the treatment in two cases, 4 series were extended to adjacent ln stations and 3 series consisted of an extended ln RT with a sequential boost. The other series were localized treatments of involved ln. For RT-planning, the gross tumor volume (GTV) was delineated via image fusion with functional imaging and a margin of 2 mm- 25 mm was applied to receive the planning target volume (PTV). The PTV was covered by the 100% isodose and dose prescriptions were according to ICRU 83 [28]. Median PTV size was 29.3 ml (Maximum 674.7 ml, Minimum 3.7 ml). In the oss-group PTV size differed between 56.2 ml and 351.9 ml (median: 72.6 ml) and was located in the pelvis (4 patients), thoracic vertebrae (2), scapula (1) or humerus (1).

**Fig. 1:**
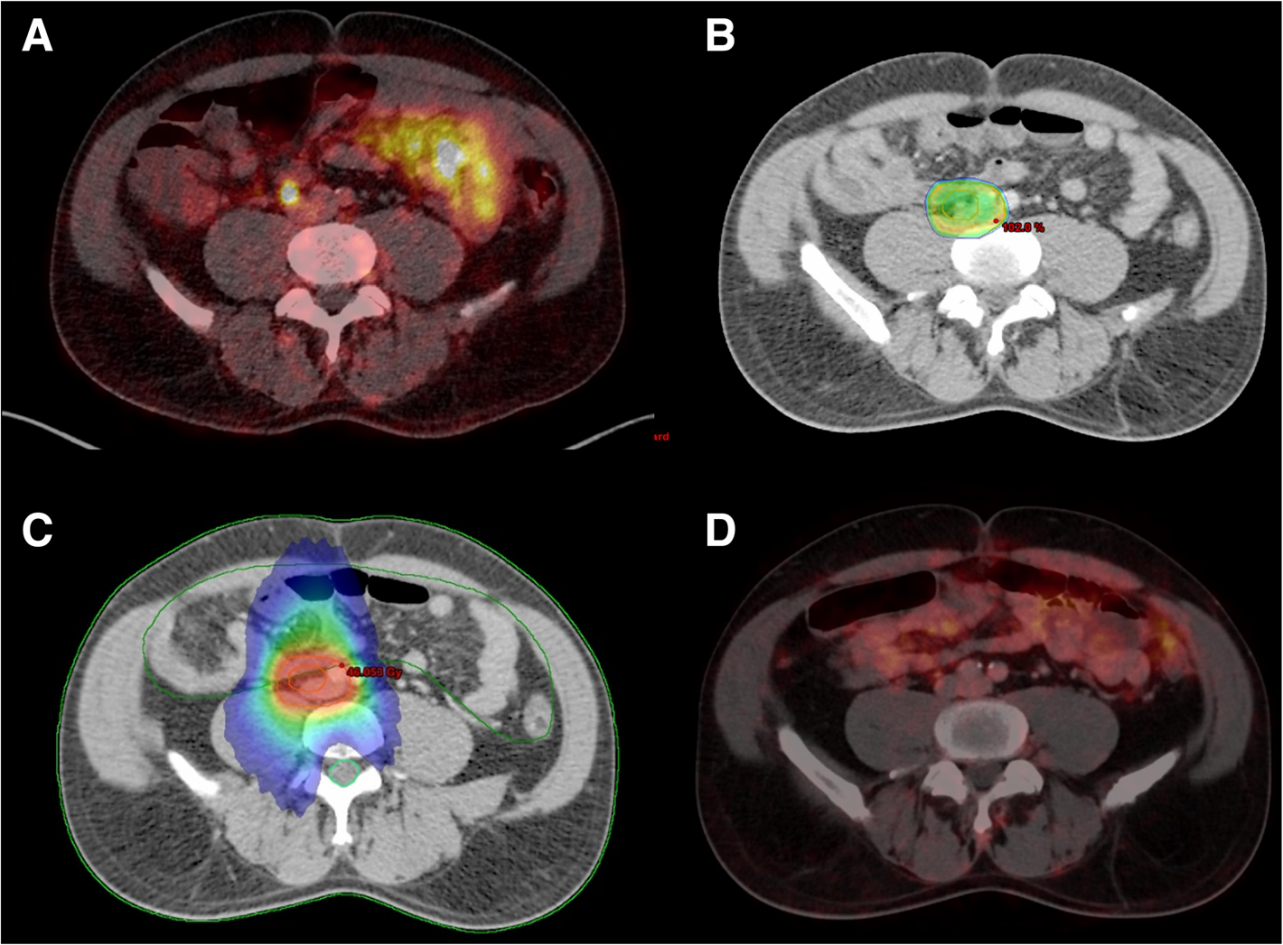
Example for ln-RT. **a** ^68^Ga-PSMA-PET-CT positive left iliacal lymph node was treated by radiation therapy up to 45 Gy and subsequent boost to 63 Gy. **b** 95%-isodose in colour-wash covering the PTV. **c** Excellent organs-at-risk sparing radiotherapy via helical tomotherapy (15 Gy isodose shown blue in colour wash). **d** No relevant tracer uptake 6 months after radiotherapy

RT was delivered as intensity-modulated and image-guided RT with a TrueBeam linear accelarator (Varian Medical Systems, Palo Alto, USA) or in helical VMAT with a tomotherapy (Tomotherapy Hi-Art II, Accuray, Sunnyvale, USA) with the usual immobilisation equipment (see Fig. 1 for an example).

In the ln-group, doses varied between 35 and 66 Gy in daily fractions of 1.8 Gy (with one exception of 7 Gy daily fraction). In 6 cases, subsequent boosts up to 70.2 Gy were applied. Median dose was 63 Gy (30.6–70.2 Gy). Comparably, doses in the oss-group were 30–66.6 Gy (median: 54 Gy) in daily normofractionated regimes of 1.8–2 Gy.

### Follow-up

Patients were seen on regular basis at their treating urologist and in the radiation oncology department with regular PSA-measurements and clinical examinations. Last follow-up was at the end of February 2018. Toxicities were graded according to the Common Terminology Criteria for Adverse Events – Score [29].

We evaluated PSA-behavior after therapy and registered a nadir, if existent, and biochemical-free survival (bfS), defined as the period of time without PSA-elevation. In addition, we evaluated metastasis-free survival (MFS) which is defined as the time after treatment without evidence of disease recurrence (on morphological/functional imaging). Overall survival (OS) was also analyzed.

### Statistics

All statistics were done with SPSS version 22 (IBM, Armok, USA). PSA-values were compared with a 1-sided Mann-Whitney-U test as a decrease in PSA was expected. OS, MFS and bfS were examined with a Kaplan-Meier-analysis, factors were compared using a log-rank-test. Correlation between RT-dose and bfS or MFS respectively were examined, using a two-sided Pearson correlation coefficient.

## Results

**Fig. 2:**
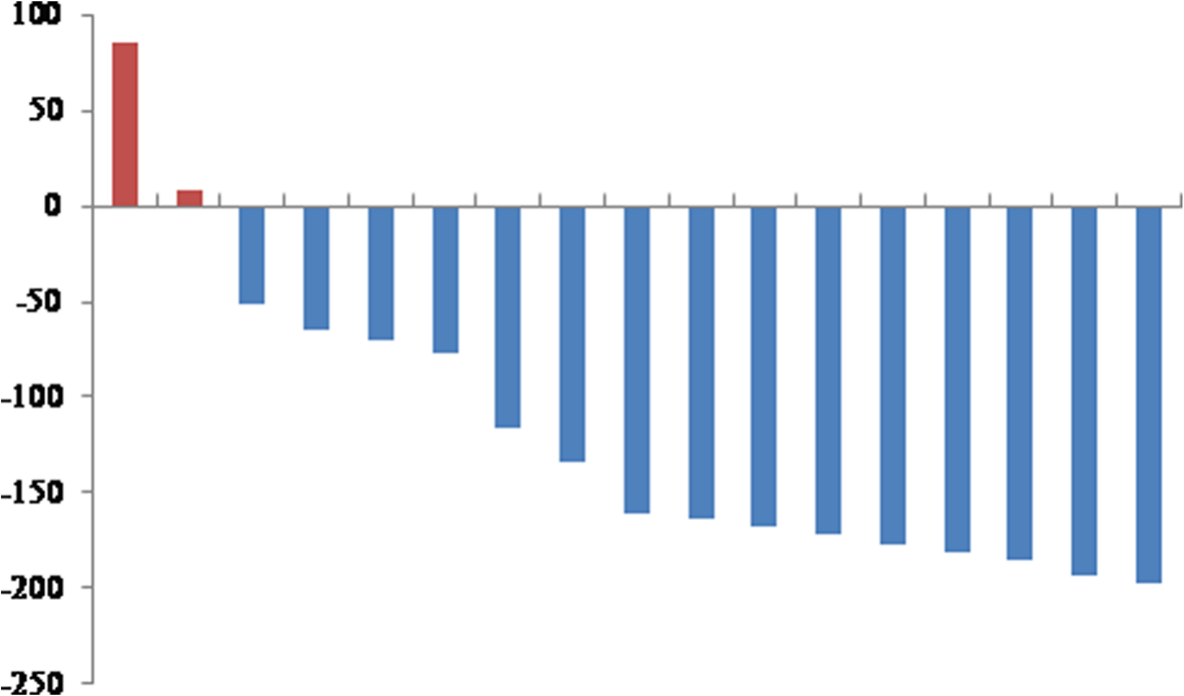
Waterfall-plot showing PSA-response to ln-RT. Each bar depicts an individual patient, the blue ones being decreases, while the two red ones indicate increase in PSA. Overall, response to therapy was > 88%

Follow-up data was available for all of the aforementioned ln-patients and mean MFS was 24.8 m (22.0–36.0 m) with a 1-year MFS of 75.4% and a 2-year MFS of 58.7% (Fig. 2a). The initial T-status (initial T2 vs. T3 carcinoma 29.3 m vs. 13.6 m; *p* = 0.013) showed an association with MFS. Neither Gleason-Score (gleason-score: 6,7 vs. higher: *p* = 0.693), number of involved ln (1–2 vs. > 2 ln; *p* = 0.544), ln-region (regionary vs. distant *p* = 0.2), initial N-status (N0 vs. N1 *p* = 0.827) or the application of concomitant ADT (*p* = 0.363) had a significant impact on MFS. There was a trend towards longer survival for R0-resected patients (*p* = 0.332) which did not reach significance. RT dose showed no linear correlation with MFS (coefficient: − 0.005; significance: 0.981).

Further analysis revealed, that morphological recurrence occurred after a mean time of 13.9 m (1.7–35.1 m). Of notice, none of these recurrences were in the RT-field, with two recurrences being adjacent to past treatment (field border), constituting a local control of 100%. There was one distant metastasis in thoracic vertebra 8, one in the 9th costa and 6 cases of additional ln-metastases, which were addressed by local RT in some cases. Four patients suffered another local recurrence after a median time of 10.7 m (3.9–41 m) with ln-metastases in 3 cases and 1 case of ln- and oss metastases.

**Fig. 3:**
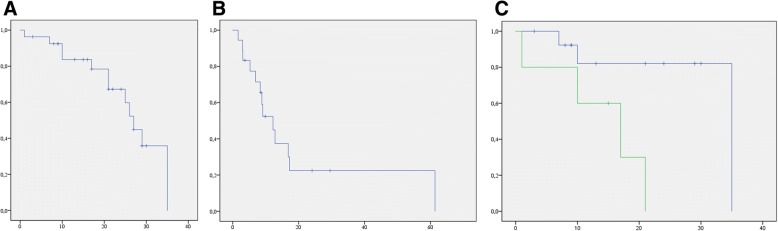
Kaplan-Meier plots showing MFS (**a**) and bFS (**b**) in months. Image **c** illustrates the different bFS for T2 (green) vs. T3 (blue) cancers in months which was significant

PSA-values were available for 17 of the 19 patients, enabling an examination of PSA-dynamics. Pre-RT median-PSA was 2.6 ng/ml (range: 0.35 ng/ml - 41.9 ng/ml), median post-RT PSA was 0.3 ng/ml (0.01–6.26 ng/ml), which was significant (*p* = 0.003). 2/17 Patients (11.8%) showed an increase in PSA, resulting in a response rate of 88.2% (see Fig. 3). Median bfS was 12.2 m (6.2–18.2 m) with a 1-year bfS of 52.4% and 2-year bfS of 22.4% (Fig. 2b). PCA which was initially confined to the prostate had a better bfS (median T2: 16.9 m, T3: 5.3 m), which was significant (*p* > 0.001; Fig. 2c). Neither the number of ln (1–2 vs. > 2; *p* = 0.955), nor the application of ADT (*p* = 0.954) had a significant impact. Pelvic ln-metastases showed a trend towards better bfS (16.9 m vs. 8.8 m), which did not reach significance (*p* = 0.082). Again, R0-resected patients tended to have a longer bfS without significance (*p* = 0.054). No significant dose-response-correlation was demonstrated between RT-dose and bfS (coefficient: − 0.074; significance: 0.385).

No patient died within the ln-group, thus overall survial (OS) was 100% with a median OS of 21.2 m (3.7–67.7 m). One patient developed secondary rectal cancer 26 m after ln-irradiation. With 3 deaths in the oss-group, mortality was higher with a median survival of 28.1 m (16.7–46.5 m).

In the oss-subgroup, all patients received ADT (1 unknown) so its impact could not be evaluated. MFS was 26 m (23.9–28.1 m) with a 1-year MFS of 100% and 2-year MFS of 83.3%. Neither T-stage (T2 vs. T3; *p* = 0.736), gleason score (6 or 7 vs. higher *p* = 0.732), tumor-free resection margin (R0 vs. R1 *p* = 0.221) had a significant impact on MFS. For PSA-follow up values were available for 6/8 patients with a median bfS of 15 m (1-year bfS 66.7%, 2-year bfS 16.7%) which was not influenced significantly by T-stage (T2 vs. T3; *p* = 0.951), gleason score (6 or 7 vs. higher *p* = 0.207), initial nodal status (N0 vs. N1 *p* = 0.277) or tumor-free resection margin (R0 vs. R1 *p* = 0.157).

There was no dose-response correlation for MFS and bfS (MFS: coefficient: − 0.234; significance: 0.288; bfS: coefficient: − 0.471; significance: 0.173). In this subcollective, 3 patients died, the mean overall survival (OS) being 38.8 m (28.8–48.9 m). Four patients revealed recurrences after a median time of 26.2 m (21.6–27.6 m) which included skeletal structures again in 4 cases with additional ln-metastasis in 1 case.

Follow-up PSA-values were available for 6 patients and revealed a median PSA-value of 4.9 ng/ml pre-treatment (range: 0.34 ng/ml – 24.8 ng/ml) which dropped to a median value of 0.14 ng/ml (0.01 ng/ml-0.68 ng/ml; *p* = 0.004). Responses were observed in 5/6 patients, thus constituting a response rate of 83.3%.

Comparing MFS and bfS between the two subcollectives, we could not find a significant difference (MFS: 26 m vs. 29 m *p* = 0.883; bfS: 15 vs. 12.2 m; *p* = 0.748).

### Toxicities

Acute toxicities were fatigue, diarrhea and increased urinary frequency. Overall, toxicities in the ln-group were tolerable with one III° toxicity of urinary incontinence, 7 II° toxicites (3 urinary incontinence, 1 urethral stricture, 1 flatulence, 1 urinary spasmus, 1 fatigue) and 7 I° toxicities (3 increased urinary frequency/nycturia, 1 diarrhea, 2 fatigue, 1 rectal bleeding). In the oss-group, toxicities were mild, as no III° toxicities or higher occurred (1 erythema I°, 1 erythema II°, 1 nausea I°, 1 fatigue, 2 increased urinary frequency/nycturia).

## Discussion

The presented study demonstrates the application of RT for oligometastatic (ln-reccurent and single oss) PCA, highlights its significant impact on PSA and identified the initial T-stadium as a significant predictor for bfS and MFS in the ln-subgroup.

To our knowledge, this was the first report of initial T-stadium as a predictor for biochemical aggressivity and treatment outcome in this clinical setting. The bfS is reduced to 5.3 m for locally advanced PCA. These PCA bear a worse prognosis à priori, although some studies showed 10-year disease-free survival of > 90% after surgical interventions [5, 6]. They also have an increased risk of ln-reccurence [30]. A possible explanation for this finding may be the distinctive and more aggressive tumor biology of advanced PCA which may be indicated by marker profile, as e.g. some isoforms of insulin-growth factor, a central signaling molecule for cellular processes, show an overexpression more frequently in advanced stages of PCA [31, 32].

Comparing different studies on RT for ln-recurrent PCA, RT-doses and -field definitions are very heterogenous. Doses may reach 74 Gy with daily fractions of up to 12 Gy [8, 9, 11, 19, 21, 23]. Hypofractionated regimes may have the potential to enhance local control [11, 19], but might not be suitable for every patient. Anyhow, RT confers a high local control of > 90% in most studies with in-field recurrences being rare events [8, 11, 21, 23]. Consistently, our study collective did not reveal in-field recurrences further encouraging the use of RT. A cut-off dosage value has been defined by Schlick et at., recommending a RT > 64 Gy for improved biochemical control [13]. Our study did not prove a significant dose-response correlation, although the biology of PCA suggests the use of high-dose regimes.

We demonstrated a 1-year MFS of 75.4% and a 2-year MFS of 58.7% while other RT-studies revealed a disease-free or clinical failure-free survival ranging from 50% after 2 y up to 61.8% after 3 y or 63.5% after 30 m respectively [8–10, 13].

Due to its retrospective nature, our patient collective includes various RT field definitions ranging from limited stereotactic setups to ln-chains with the inclusion of the former prostate bed in 2 cases, which also reflects the various concepts described by other authors [8–11, 13, 20]. Of importance, even an extended whole-pelvic RT does not exclude further metastatic spread [8] while the idea of limited toxicity with a small field delineation is not necessarily corroborated by the literature [10, 11, 13]. Thus, the field and dose decisions demand further investigation.

Local RT directed towards ln metastases also influence PSA significantly, a known effect [8–11, 13]. The use of ADT in the literature as well as in the majority of our study patients prompts the evaluation of PSA-dynamics rather than absolute values. Overall, PSA-response rate to treatment was 88.2%, which is comparable to previous studies (81–91.4%) [9, 11, 20]. Anyhow, some studies point out a possible “delay” of ADT up to 44 m till the beginning of ADT by treating oligometastases with RT [21, 25].

During and after treatment, we could demonstrate an excellent toxicity profile with expected side effects for pelvic irradiation withour any III° or higher toxicites. Of notice, the patients in our collective were extensively pretreated and were in a (re-)salvage situation.

The location of ln-metastases may have a prognostic impact as suggested by the study of Fodor et al. who identified extrapelvic ln metastases as a significant factor for worse OS [9]. As no patient died in our study, we could not examine this parameter, but MFS may be a suitable surrogate. Although not being significant, there was a trend towards better MFS and bfS for regionary metastases. This finding may encourage the use of local therapies like pelvic radiation or ln-dissection for PCA confined to pelvis as they may enable a long disease-free survival. In contrast, distant-ln metastases warrant an intensified, systemic therapy. Interestingly, a tumor positive resection margin may herald a worse MFS, which may be explained by the lack of local control in the pelvis.

A deterioration in prognosis is found with increasing number of metastases, the cutoff number being controversial (1–3) [33–36]. In our study collective, the number of ln was not a significant predictor for MFS or bfS in comparison between 1 and 2 or > 2 ln. Nevertheless, our patients had a maximum of 5 ln-metastases (thus making them suitable candidates for RT) and true polymetastatic patients were not included. Importantly, the analysis by Schweitzer et al. was not limited to ln-metastases and Briganti et al. focused on patients with ln-metastases in the primary therapy [33, 36], which contrasts the initially predominant N0-patients in our study. These studies underline a superiority in prognosis for mono- or oligometastatic disease.

The lack of a significant impact of R-status and ln-number on bfS may be caused by the small study collective as both parameters showed a trend towards better bfS in the more limited categories. This may reflect the increased risk of local and biochemical failure for positive resection margins in the primary therapy [37, 38].

It has to be emphasized, that diagnosis relies essentially on functional imaging. With the advent of Gallium as a PMSA PET-Tracer, sensitivity especially at low PSA-levels has increased remarkably [39]. Most studies in the literature were conducted with a choline-tracer [9, 11, 12, 20, 35] which demonstrates inferiority in PCA detection, especially at a PSA < 2 ng/ml [39]. The meta-analysis by von Eyben et al. revealed a detection rate of 50% for recurrent PCA in men with a PSA as low as 0.2 ng/ml [22], thus enabling an image-guided therapy as in our study. A recent study on ^68^Ga-PSMA PET-scans used in patients with biochemical-relapse after radical prostatectomy demonstrated that metastases were confined to the prostate bed in only 30.3% independent of PSA-value, whereas a PSA > 1 ng/ml was significantly associated with extrapelvic recurrence [40]. Despite increasing sensitivity, false-negative patients who do not undergo a “targeted” local approach via surgery or RT but only receive ADT have to be considered. In case of serious doubts, repetitive imaging in the short-term follow-up may be suitable.

RT has an established role in the treatment of oss-metastases [5, 6] but may experience a renaissance in the context of oligometastases with putative curative intent, an indication not covered by national and international guidelines so far [23, 27]. We decided to include only patients with solitary bony metastases as increasing number of metastases may herald a worse survival [41]. In a study with 15 patients (20 lesions) using hypofractionated concepts (5 fractions 5–7 Gy with additional simultaneous boost in some cases) progression-free survival was 7.4 m with a high local control (only 1 failure) and a slight increase in the time till ADT onset/intensification of systemic therapy [18]. PSA-progression-free survival was 6.9 m but only 3 patients in this study received ADT [18], hampering a direct comparison with our data.

The hereby presented study is the first to demonstrate the equality of MFS and bFS for ln- and oss-category. In a comparative study including ln- as well as oss-oligometastases, Schick et al. found a 3-year bfS of 54.5% and an OS of 91.7% taking both categories alltogether [13]. In accordance with that, a Belgian study group investigated the subject with no direct oss- and ln- comparison [23]. Interestingly, it could be demonstrated that relapses of ln-metastases are found mainly in ln, while oss-metastatic patients relapse predo-minantly in the bone [23], a finding also corroborated in the hereby presented study. The same research group conducted a prospective phase II-trial in which metastases-directed therapy (mostly RT) showed a clear advantage in bFS further postponing ADT-onset in comparison to the observation group while maintaining an excellent toxicity profile without II°-toxicities [24].

Our study bears several shortcomings as patient number and follow-up are yet limited. Furthermore, it is retrospective and monocentric, but already points out important aspects for the use of RT in this clinical setting.

Further studies are warranted: As ADT was utilized in most cases of our study, its precise impact may not be evaluated and it remains state-of-the art treatment for ln-recurrent PCA. Notwithstanding this fact, RT and ADT are not to be seen as mere competitors but may have synergistic effects encouraging a combined use, with ADT controlling PCA systemically and RT conferring a high local control, a possibility pointed out by the literature [13] and our study. Another combined modality treatment is the combination of a ln-dissection and RT for ln-recurrences further enhancing local control and relapse free-survival [42]. Ongoing studies (www.clinicaltrials.gov, search on: 6th august 2017: NCT01859221, NCT02274779, NCT02563691, NCT02680587, NCT02685397, NCT02759783, NCT02816983) investigate the role of RT in an oligometastatic setting. The prospective phase II trial NCT02274779 (OLIGOPELVIS – GETUG P07) evaluates the use of RT for 1–5 pelvic nodal metastases in combination with an ADT concerning bfS and relapse-free survival [43]. Patient enrollment has ended and final results are expected in autumn/winter 2018 [43]. New insights into the biology of PCA and its possible and necessary treatment options are expected.

## Conclusions

Our study points out the feasibility of RT as a treatment option in ln recurrent PCA. It has demonstrated an excellent local control with a low-toxicity profile and may complement androgen-deprivation therapy as a local treatment option.

## Abbreviations

ADT: Androgen-deprivation-therapy
bfS: Biochemical-free survival
DRE: Digital-rectal exam
GTV: Gross tumor volume
Ln: Lymph node
m: Month
MFS: Metastasis-free survival
OS: Overall survival
oss: Single-osseous
PCA: Prostate cancer
PSA: Prostate-specific antigen
PSMA: Prostate-specific membrane antigen
PTV: Planning target volume
RT: Radiotherapy
VMAT: Volumetric arc therapy
y: year
